# Preoperative multidetector-row computed tomography scan staging for lymphatic gastric cancer spread

**DOI:** 10.1186/1477-7819-10-197

**Published:** 2012-09-24

**Authors:** Paolo Morgagni, Enrico Petrella, Barbara Basile, Alberto Mami, Augusto Soro, Andrea Gardini, Filippo Calzolari, Domenico Garcea, Mauro Bertocco

**Affiliations:** 1 Department of General Surgery, Morgagni-Pierantoni Hospital, Via Forlanini 34, Forlì, Italy; 2 Radiology Unit, Morgagni-Pierantoni Hospital, Via Forlanini 34, Forlì, Italy

**Keywords:** MDCT staging, Gastric cancer, Lymph-node diffusion, Preoperative setting

## Abstract

**Background:**

Multidetector-row computed tomography (MDCT) is commonly used to stage patients with gastric cancer, even though the technique often shows low specificity for lymph-node involvement.

**Methods:**

In this study, 111 patients with gastric cancer who consecutively underwent MDCT scan followed by radical surgical treatment at our hospital were retrospectively evaluated.

**Results:**

In total, 3632 lymph nodes from 643 lymphatic stations were studied and then correlated with radiological features. Lymph-node size was not always associated with infiltration. Of the 261 lymph-node stations that were not radiologically detected, 60 (22.9%) were infiltrated. There were 108 stations with lymph nodes larger than 10 mm seen on MDCT, of which 67 (62%) had lymphatic invasion. The sensitivity was 32.6%, specificity 90.6%, positive predictive value 62.0%, negative predictive value 74.2%, and accuracy 72.1%. When three lymph nodes, at least one of which was larger than 10 mm, were detected in the same station, infiltration was confirmed with 99% specificity in 93.8% of patients. Moreover, all of the 13 patients in whom three lymph nodes larger than 10 mm were detected in different neighboring stations had lymphatic invasion.

**Conclusions:**

Although presence of lymph nodes greater than 10 mm in size is not, in itself, sufficient to confirm lymphatic invasion, nodal involvement can be hypothesized when associated images are detected by MDCT.

## Background

With the exception of early lesions, gastric cancer is generally considered a tumor with a poor prognosis, and surgical treatment alone does not offer great hope to patients with serosal involvement or lymphatic diffusion. Given that neoadjuvant treatments are currently proposed for advanced cancer, the preoperative stage of the tumor must be determined first in order to avoid using inappropriate medical treatment in patients who are potentially radically treatable by endoscopic or surgical therapy. Although improvements in endoscopic ultrasonography are continuously being made in terms of defining cancer infiltration, the accuracy of this method in identifying suspect non-perigastric lymph-node involvement and metastases remains poor.

One of the most widely used diagnostic methods for staging of these patients is multidetector-row computed tomography (MDCT) [1-3], which has high sensitivity in identifying distant metastases or enlarged lymph nodes, but is often inadequate in recognizing lymph-node metastasis. Although lymph nodes larger than 10 mm in size are generally considered to be positive, other criteria for identifying involved nodes have been reported in the literature, including a size of greater than 6 mm plus round shape; size of greater than 8 mm on the short axis, size of greater than 8 mm irrespective of axis; or simply radiologically detection of the node [1]. The concept of bulky lymph nodes has emerged from literature data to define a high suspicion of malignant lymph-node infiltration when lymph nodes are greater than 30 mm in size or there are more than three lymph nodes measuring 15 mm each present in neighboring stations [3].

The aim of this study was to correlate lymphatic size and infiltration in our patients, and to verify whether 16-row MDCT could effectively help to identify patients with node-positive cancer for neoadjuvant treatment.

## Methods

### Ethics approval

As MDCT scan and lymphatic dissection were considered the standard treatment for all our patients, ethics approval was not needed and only informed written consent was obtained.

### Patients and treatment

From January 2009 to January 2011, we analyzed 111 patients with gastric cancer at Morgagni-Pierantoni Hospital in Forlì, who had consecutively undergone 16-row MDCT scan followed by radically treatment. During this period, a standard approach for MDCT scan was used by the radiology unit of our hospital for cases of suspected gastric cancer. Specifically, after a 12-hour fast, patients were required to drink 600 ml of water and then lie down in a supine position (for gastric cancer of the antrum-corpus) or a prone position (for gastric cancer of the upper third of the tract). Patients were give 20 mg of scopolamine N-butyl bromide (Buscopan®; Boehringer Ingelheim, Tokyo, Japan) as an intravenous bolus infusion. The technical parameters required are described in Table 1.

**Table 1:** Technical parameters required for multidetector-row computed tomography

**Pre-contrast phase thickness**	**3.75 mm**
Arterial phase thickness	2.5 mm
Portal phase thickness	2.5 mm
Pitch	1.25
Rotation time	0.5 seconds
Contrast	2 ml/kg at 3.5 ml/second
	Arterial phase with smart prep, venous phase at 70 seconds	
Reconstructions on sagittal and coronal planes		

The results from all MDCT scans were revised for this study by five radiologists in order to accurately determine lymph-node shape and size along the major axis in the area of the stations dissected by surgeons. Lymph-node size was arbitrarily subdivided into five categories (major axis <5 mm, 5 to <10 mm, 10 to <15 mm, 15 to <20 mm, and ≥20 mm). All radiologically detected lymph nodes were separately registered for each lymphatic station in accordance with Japanese Gastric Cancer Association (JGCA) recommendations [4], with special attention being paid to associations of enlarged lymph nodes such as three lymph nodes in the same station or presence of enlarged lymph nodes in nearby stations. Dissected stations where only fatty tissue was identified (as frequently occurs in the suprapyloric station) were not considered for this study.

Subtotal gastrectomy was then performed for tumors sited in the lower two-thirds of the stomach, whereas total gastrectomy without standard splenectomy was carried out for tumors in the upper third of the stomach. Both interventions were completed by level I or II lymphadenectomy (D1 or D2), based on the age and condition of the patient [4], and removal of the greater and lesser omentum. All lymphatic stations were dissected by surgeons immediately after resection for separate histological examination. Tumors were classified in accordance with the Union for International Cancer Control tumor, node, metastasis (UICC TNM) classification (seventh edition) [5].

We then correlated the shape and size of radiologically detected lymph nodes in each lymphatic station with the histological diagnosis of any lymph nodes dissected in that station. To assess the correlation between the size of MDCT-detected lymph nodes and infiltration, stations were considered radiologically positive if at least one lymph node was detected by imaging in that area, and histologically involved if at least one of the separately dissected lymph nodes was infiltrated. Clinical, radiologic, and pathologic data were stored in the database. Sensitivity, specificity, positive and negative predictive value (PPV and NPV), accuracy, and likelihood of lymph-node involvement were determined to evaluate diagnostic efficiency.

## Results

The characteristics of the 111 patients are summarized in Table 2. In total, 3632 lymph nodes from 643 stations were dissected (median 32.7 lymph nodes/patient). There were 832 lymph nodes detected by our radiologists in 382 stations; 75 stations had lymph nodes smaller than 5 mm in size, whereas 83 had lymph nodes that were 10 mm or larger. Only three stations with lymph nodes larger than 20 mm were seen in our series.

**Table 2:** Patient characteristics

**Patient characteristics**	***P*atients**	
	**n**	**%**
Sex		
Male	57	51.3
Female	54	48.7
Histology		
Intestinal	89	80.2
Diffuse mix	22	19.8
T staging		
T1a	17	15.3
T1b	14	12.6
T2	6	5.4
T3	23	20.7
T4a	46	41.5
T4b	5	4.5
N staging		
N0	44	39.6
N1	15	13.5
N2	14	12.6
N3a	16	14.5
N3b	22	19.8
Tumor site		
Upper third	28	25.2
Middle third	28	25.2
Lower third	55	49.6
Tumor size, mm		
≤2	14	12.6
>2 to ≤4	38	34.2
>4	59	53.2
Lymph-node dissection		
D1	9	8.1
D2	102	91.9

**Figure 1:**
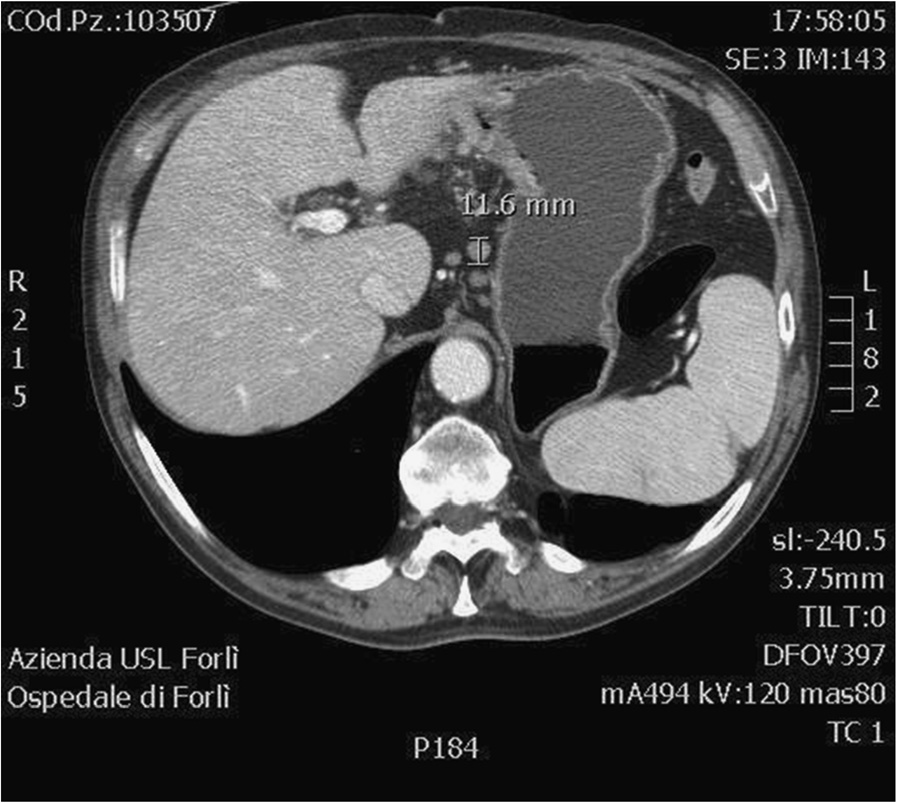
Three positive lymph nodes were detected in the same station, one of which was larger than 10 mm.

Of the 382 MDCT-detected lymph-node stations, 237 were histologically negative and 145 were infiltrated. Correlations between radiological size and pathological station involvement are summarized in Table 3. Although the percentage of involved stations rose as lymph-node size increased, low sensitivity and specificity (Table 4). In particular, only 22.9% of the 261 stations not radiologically detected were infiltrated; taking into consideration radiologically detected lymph nodes, 16% of the stations with nodes smaller than 5 mm, 33% of the stations with nodes between 5 and 10 mm, and 63% of stations with nodes between 15 and 20 mm were histologically infiltrated. Conversely, when the association of lymph nodes was analyzed, a higher percentage of positive lymph nodes was detected when there were three or more lymph nodes in the same station and one of these was larger than 10 mm in size (Figure 1). Stations with these characteristics were positive in 93.8% of cases and, although sensitivity was low, specificity was high. Furthermore, associations of three lymph nodes larger than 10 mm in neighboring stations always identified patients with lymphatic involvement (Table 5). Taking lymph-node size into consideration, the sensitivity fell and specificity rose as size increased.

**Table 3:** Correlation between radiological size and clinical characteristics

**Lymph nodes**	**Total stations, n**	**Positive stations, n**	**Total stations/positive stations, %**
Dissected but not radiologically detected	261	60	22.9
Size, mm			
<5	75	12	16
≥5 to <10	199	66	33.1
≥10 to <15	83	50	60.2
≥15 to <20	22	14	63.6
≥20	3	3	100

**Table 4:** Correlation between radiological size of the largest lymph node per station and sensitivity, specificity, predictive value, accuracy, and likelihood ratio

**Lymph nodes**	**Radiologically detected**	**Pathologically infiltrated**	**Sensitivity**	**Specificity**	**PPV**	**NPV**	**Accuracy**	**LR (95% CI)**
All radiologically detected lymph nodes	382	145	70.7	45.8	37.9	77	53.8	1.30 (1.15 to 1.47)
Size, mm								
<5	307	133	64.8	60.2	43.3	78.5	61.7	1.63 (1.40 to 1.90)
≥10	108	67	32.6	90.6	62.0	74.2	72.1	3.49 (2.45 to 4.96)
≥15	25	17	6.8	98.1	63.6	69.2	69	3.73 (1.59-8.77)
≥20	3	3	1.4	100	100	68.4	68.5	Not measurable

LR, likelihood ratio; NPV, negative predictive value; PPV, positive predictive value.

**Table 5:** Size of lymph nodes and station involvement when associated lymph nodes were reported; sensitivity, specificity, predictive value, accuracy and likelihood ratio

**Characteristic**	**Radiologically detected**	**Pathologically infiltrated**	**Sensitivity**	**Specificity**	**PPV**	**NPV**	**Accuracy**	**LR (95% CI)**
Stations with ≥3 lymph nodes radiologically detected, one of which was >10 mm	49	46	22.4	99.3	93.8	88.0	74.8	32.7 (10.31 to 104.0)
Patients with >3 lymph nodes >10 mm in neighboring stations	13	13	6.3	100	100	69.5	70.1	Not measurable

LR, likelihood ratio; NPV, negative predictive value; PPV, positive predictive value.

PPV, which was lower in non-radiologically detected lymph nodes (37.9%), increased to 100% when lymph nodes larger than 20 mm were present. NPV decreased from 77% to 68.4% and accuracy increased from 53.8 to 68.4%. Sensitivity was very low, but specificity and PPV were high when the two associations of lymph nodes previously described were taken into consideration (Table 5). The likelihood ratio (LR) also increased as lymph-node size increased, but was very high in patients with one lymph node larger than 10 mm and two radiologically detected lymph nodes in the same station. The LR for these data was 32.7 (95% CI 10.3 to 104)

With regard to lymph-node shape, 46.1% of overall lymph nodes were round and 53.9% were oval while 49.3% of positive dissected lymph nodes were round and 50.7% were oval.

## Discussion

In recent years, increased interest in integrated treatments, especially in a neoadjuvant setting, has led to more accurate preoperative staging and to better selection of patients who are candidates for more specific treatment.

Although MDCT scan is not considered the gold standard technique for T and perigastric N staging (sensitivity and specificity range from 60% to 90%) [1,6], it is nevertheless the most widely used standard diagnostic tool. Conversely, echo-endoscopy, considered by several authors as the most accurate method to study tumor growth and perigastric nodes (median sensitivity and specificity values reported in the literature of 70.8% and 84.6%, respectively, or 95.3% and 100% in selected centers) [1], is not always performed, and does not have a high level of accuracy in identifying distant lymph nodes. This problem is further increased by the new TNM staging system, which requires the selective identification of single lymph nodes to differentiate between N1, N2, and N3 stages. Numerous studies have been published on the correlation between lymph-node dimensions and neoplastic infiltration, and a size of 10 mm is generally considered to have a high degree of suspicion for infiltration [2]. Different criteria have been proposed for defining metastatic infiltration, including size of 6 mm along the short axis diameter of perigastric lymph nodes [7]; 8 mm along the short [8] or greater axis [9]; or size associated with other characteristics such as marked enhancement [2], necrosis [7], shape [8] or fat content [7,10,11]. Other authors consider all identifiable lymph nodes to be positive [1,12].

Several studies have estimated the N radiological stage by taking into consideration the distance between lymph node and tumor [7,12] (old TNM) or lymphatic station level, as reported by the JGCA [8], but few studies have considered single lymph-node stations [13]. In the current study, it was not possible for us to study lymph nodes separately, and we therefore evaluated single stations dissected by the surgeon at the end of the surgical treatment. Our data failed to confirm a close correlation between size and infiltration, although increased lymph node size was more frequently associated with metastasis. In particular, metastases were found in 22.9% of undetected lymph nodes and only in about 60% of lymph nodes 10 mm in size. Although lymph nodes larger than 20 mm were always infiltrated, few were identified and all showed low sensitivity. Furthermore, shape alone was not a specific criterion to define infiltration, with at least 50% of the detected lymph nodes being oval-shaped.

Yoshikawa et al. defined ‘bulky lymph node metastasis’ as one node of 30 mm or larger in diameter, or at least three consecutive nodes each 15 mm or larger in first- or second-level lymph-node stations [3]. Although we did not observe any lymph nodes larger than 30 mm, and very few patients had an association of three lymph nodes of 15 mm or more in size, we found that the association of three lymph nodes, one of which larger than 10 mm, was generally associated with infiltration, with 49 stations having this characteristic, and infiltration being detected in 46 of these. Only one of the two patients who did not show any correlation was staged N0, whereas the other was staged N3. The presence of lymph nodes larger than 10 mm in neighboring stations was seen in 13 patients, all of whom had lymphatic involvement, even though some large lymph nodes analyzed were histologically negative (Table 5). Even though sensitivity was low and few patients were involved, we believe that these two lymph-node correlations could be important criteria to select individuals with lymphatic spread because of the high specificity (almost 100%).

## Conclusions

MDCT scans are widely used in preoperative gastric cancer staging. In this study, perigastric lymph-node dimensions showed low sensitivity and higher specificity as node size increased, and evaluation of larger lymph nodes located in the same area led to increased accuracy. Because sensitivity, specificity, and accuracy of lymph-node dimensions alone would not seem to predict lymph-node infiltration, further research is needed to find correlations between node size and other characteristics in order to guarantee adequate preoperative staging. Although we only analyzed a small number of patients, the correlation found between the number of lymph nodes and their size is interesting and, if supported by larger, statistically analyzed studies, could help to identify patients who are candidates for preoperative treatment.

## Competing interests

The authors declare that they have no competing interests.

## Authors’ contributions

PM, BB, EP, and DG were involved in study conception and design; EP, AM, FC, BB, PM, and AS carried out data acquisition; EP, AM, FC, BB, PM, and AG were responsible for quality control of data and algorithms; AG and PM carried out data analysis and interpretation; PM, DG and FC drafted the manuscript. All authors read and approved the final manuscript.
